# Stem Cell-Based Therapy in Transplantation and Immune-Mediated Diseases

**DOI:** 10.1155/2017/7379136

**Published:** 2017-09-06

**Authors:** Vladislav Volarevic, Majlinda Lako, Slaven Erceg, Miodrag Stojkovic

**Affiliations:** ^1^Faculty of Medical Sciences, Department of Microbiology and Immunology, Center for Molecular Medicine and Stem Cell Research, University of Kragujevac, Kragujevac, Serbia; ^2^Institute of Genetic Medicine, Newcastle University, Newcastle, UK; ^3^Principe Felipe Research Center, Valencia, Spain; ^4^Faculty of Medical Sciences, Department of Genetics, University of Kragujevac, Kragujevac, Serbia; ^5^Spebo Medical, Leskovac, Serbia

Despite the enormous scientific progress in the field of regenerative medicine, there are no cells that provide a cure for everything and everyone. Stem cells have raised tremendous expectations among the medical doctors, researchers, patients, and the general public due to their capacity to differentiate into a broad range of cell types. Stem cell researchers are engaged in different endeavors, including treating genetic disorders and generating new stem cell-derived human tissues and biomaterials for use in pharmacy, genomics, and regenerative medicine. Results obtained from completed and on-going clinical studies during the last decades demonstrate rapid progress and the increased importance of stem cells in both basic research and the long-term future of modern medicine indicating the huge therapeutic potential of stem cells in the treatment of degenerative, autoimmune, and genetic disorders.

Mesenchymal stem cells (MSCs) are considered as new therapeutic agents in the treatment of immune-mediated diseases, particularly due to their immunomodulatory characteristics. In cell-to-cell contact and through the production of soluble mediators, MSCs can regulate the proliferation, activation, and effector function of T lymphocytes, professional antigen presenting cells, NK cells, NKT cells, and neutrophils.

MSCs suppress inflammatory (M1) macrophages and promote their conversion in alternative (M2) phenotype in prostaglandin E2 (PGE2), tumor necrosis factor alpha- (TNF-*α*-) stimulated gene/protein 6 (TSG-6), interleukin- (IL-) 6, and indoleamine 2,3-dioxygenase- (IDO-) dependent manner. In line with these findings, in their article published in this special issue, S. Ravanidis and coworkers described molecular mechanisms involved in interplay between macrophages and multipotent adult progenitor cells (MAPC), a stem cell population sharing common mesodermal origin with MSCs. S. Ravanidis et al. showed that MAPC, in a cyclooxygenase 2- (COX-2-) dependent manner, suppressed the secretion of TNF-*α* in M1 inflammatory macrophages while, at the same time, inflammatory macrophages triggered the immunomodulatory properties of MAPC, including an increased expression of immunomodulatory mediators (inducible nitric oxide synthase (iNOS) and COX-2), chemokines, and chemokine receptors. Moreover, S. Ravanidis and colleagues showed that the MAPC secretome suppressed the antigen-specific proliferation of autoreactive T cells and attenuated their capacity to activate inflammatory macrophages. Data published in this article revealed mechanisms involved in the interactions between MAPC and inflammatory macrophages, which could be important for the design of new MAPC-based therapeutic strategies for the treatment of inflammatory disorders in which myeloid cells play a crucial role.

MSCs are adult stem cells that can be isolated from various numbers of postnatal tissues. Bone marrow (BM) has been the main source for the isolation of multipotent MSCs. BM-MSCs have many properties that enable their therapeutic use: easy acquisition, quick proliferation *in vitro*, low surface expression of major histocompatibility complex (MHC) antigens and minor immunological rejection, long-term coexistence in the host, maintenance of differentiation potential after repeated passages, and ease of transplantation. Since the derivation of BM-MSCs involves harvesting of BM that is a highly invasive procedure, alternative sources have been strongly pursued. In this special issue, immunomodulatory characteristics of BM-MSC, synovial-membrane-derived MSCs (SM-MSCs), human placenta-derived MSCs (hP-MSCs), and adipose tissue-derived MSCs (AT-MSCs) were described and their therapeutic potential was investigated in well-established animal models of acute renal injury, arthritis, and liver fibrosis.

Since AT-MSCs can be easily isolated from liposuctions, AT represents an important source of MSCs. Accordingly, Y. Wu and coworkers investigated cytokine secretion profile of AT-MSCs in order to further emphasize their potential in cellular immunotherapy. As it was described by Y. Wu et al., AT-MSC cultures were heterogeneous in phenotype indicating that all AT-MSCs did not contribute to the immune-suppression in the same manner. Although all AT-MSCs were capable to produce IL-6, only 10–20% of AT-MSCs expressed IL-6 receptor (CD130) on their surface. Similarly, IFN-*γ* was produced by only 1.4% of AT-MSCs, but 18–31% of AT-MSCs expressed receptor of IFN-*γ* (CD119) indicating that AT-MSCs did not produce inflammatory cytokines but had the capacity to respond on them. Results obtained by Y. Wu and colleagues demonstrate that AT-MSCs are heterogeneous in their cytokine secretion and receptor expression profiles which are an important information for their future therapeutic use.

AT-MSCs were also in the focus of the research conducted by I. Muñoz-Criado et al. who investigated regenerative potential of AT-MSCs in the animal model of severe osteoarthritis (OA). They showed that transplantation of suprapatellar-derived AT-MSCs significantly diminished the OA-associated knee inflammation and cartilage degenerative grade by increasing the production of glycosaminoglycan and by inducing endogenous chondrogenesis. Results obtained by I. Muñoz-Criado and colleagues strongly suggest transplantation of autologous suprapatellar-derived AT-MSCs as a new therapeutic approach for patients with multiple degenerative OA.

Advanced liver fibrosis results in cirrhosis, liver failure, and portal hypertension and often requires liver transplantation. However, liver transplantation has several limitations, including lack of donors, complications of surgical interventions, side effects of immuno-suppressive drugs, and high medical costs. Accordingly, the alternative approaches such as stem cell transplantation have been suggested as an effective alternate therapy to liver transplantation. In line with these findings, J. Yu and coworkers showed that transplantation of hP-MSCs efficiently repaired carbon tetrachloride- (CCl_4_-) induced liver fibrosis in rats, as evaluated by enhanced liver function tests, improved histopathology, reduced Sirius red-stained collagen area, and downregulated expression of fibrotic markers: transforming growth factor beta (TGF-*β*) and alpha smooth muscle actin (*α*-SMA). Moreover, by using green fluorescent protein, J. Yu et al. demonstrated that transplanted hP-MSCs successfully engrafted in the injured livers where it were able to restore liver function, suggesting their therapeutic potential in the treatment of liver fibrosis.

In many cisplatin-treated patients, kidney injury is irreversible, requiring substitution, reduction, or discontinuation of cisplatin treatment. Since currently there is no compatible and convenient chemotherapeutic agent with similar potent, anticancer efficacy as cisplatin, clinical use of cisplatin cannot be abandoned. Accordingly, an urgent demand exists for researchers to develop new adjuvant therapy for attenuation of cisplatin-induced nephrotoxicity and inflammation. B. S. Markovic and coworkers demonstrated that BM-MSCs can attenuate cisplatin-induced acute renal failure by suppressing migration and activation of immune cells in inducible nitric oxide synthase- (iNOS-) dependent manner. They noticed significantly decreased levels of inflammatory cytokines TNF-*α* and IL-17 and increased levels of anti-inflammatory cytokines IL-10, IL-6, nitric oxide (NO), and kynurenine in sera of cisplatin-treated mice that received BM-MSCs or MSC-conditioned medium (MSC-CM) indicating that MSCs exert their beneficial effects in paracrine manner. Moreover, BM-MSC or MSC-CM treatment significantly attenuated influx of immune cells: macrophages, dendritic cells, neutrophils, and T lymphocytes in damaged kidneys and attenuated their capacity to produce TNF-*α* and IL-17. Importantly, inhibition of iNOS completely diminished renoprotective and immuno-suppressive effects of MSC-CM. Results obtained by B. S. Markovic and colleagues provide the evidence that BM-MSCs, in paracrine, iNOS-dependent manner, attenuate inflammation in cisplatin-induced nephrotoxicity. These findings could be helpful in developing new BM-MSC-based therapeutic approaches for attenuation of cisplatin-induced nephrotoxicity.

Rheumatoid arthritis is an autoimmune, systemic inflammatory disease characterized by persistent inflammation, extensive synovial hyperplasia, and cartilage and bone destruction that is developed as a consequence of enhanced Th1 and Th17 immune response and suppressed activity of T regulatory and B regulatory cells. MSCs have the potential to suppress both Th1- and Th17-driven inflammation and to promote expansion of regulatory cells in peripheral lymph organs. Accordingly, in this special issue, M. Yan and coworkers demonstrated that intra-articular injection of SM-MSCs ameliorated clinical and histological severity of collagen-induced arthritis by decreasing production of Th1 and Th17 inflammatory cytokines (TNF-*α*, IFN-*γ*, and IL-17A) and by increasing production of anti-inflammatory and immuno-suppressive IL-10. Moreover, cellular makeup of the spleens revealed decreased number of Th1 and Th17 cells and increased presence of Th2 lymphocytes, PD-1+CXCR5+FoxP3+ follicular T regulatory cells, and CD19+CD5+CD1d+IL-10+ regulatory B cells in mice that received SM-MSCs. Data obtained by M. Yan and colleagues demonstrated therapeutic potential of SM-MSCs for the suppression of immune response and inflammation during the progression and development of rheumatoid arthritis.

Although immuno-suppressive characteristics of MSCs are beneficial in the treatment of inflammatory and autoimmune diseases, they can represent a serious problem if patient that received MSCs has primary or metastatic tumor. By using mice model of metastatic lung cancer, M. Gazdic and coworkers showed that intravenous application of BM-MSCs significantly suppressed systemic antitumor immune response, reduced total number of lung-infiltrated dendritic cells, macrophages, and CD4+ T lymphocytes, and attenuated antitumor cytotoxicity of cytotoxic T lymphocytes and NK cells resulting with the expansion of metastatic lesions in the lungs. This phenomenon was abrogated by inhibitors of iNOS and IDO, suggesting importance of iNOS and IDO for MSC-mediated suppression of antitumor immune response. Data obtained by M. Gazdic and colleagues raise serious concerns regarding safety of MSC-based therapy in patients who have genetic susceptibility for malignant diseases.

In summing up, articles in this special issue present novel findings regarding molecular and cellular mechanisms involved in MSC-based suppression of immune response in inflammatory and malignant diseases underlining the importance of preclinical studies in the development of efficient and low-cost regenerative medicine.

*Vladislav Volarevic* *Majlinda Lako* *Slaven Erceg* *Miodrag Stojkovic*

